# F-actin cytoskeleton reorganization is associated with hepatic stellate cell activation

**DOI:** 10.3892/mmr.2014.2036

**Published:** 2014-03-11

**Authors:** XIAODONG CUI, XIAOYUN ZHANG, QINGLING YIN, AIXIA MENG, SHAOJUAN SU, XU JING, HONG LI, XIUMEI GUAN, XIN LI, SHUNMEI LIU, MIN CHENG

**Affiliations:** Medical Research Center, Weifang Medical University, Weifang, Shandong 261053, P.R. China

**Keywords:** hepatic stellate cells, F-actin, cytoskeleton, migration, adhesion, gene expression

## Abstract

The activation of hepatic stellate cells (HSCs) is involved in the development of hepatic fibrosis. Previous studies have indicated that the acquisition of certain properties by activated HSCs is highly dependent on the reorganization of the actin cytoskeleton. However, direct evidence showing that the reorganization of the actin cytoskeleton is responsible for HSC activation is lacking. The aim of the present study was to investigate the role of cytoskeletal reorganization during HSC activation and to clarify the underlying mechanism. HSC-T6 cells were treated either with the F-actin stabilizer jasplakinolide (Jas) or the depolymerizer cytochalasin D (Cyto D). The actin cytoskeleton was evaluated via assessment of stress fiber formation. Furthermore, the activation properties of HSCs, including proliferation, adhesion, migration and the expression of α-smooth muscle actin (α-SMA) and collagen 1, were investigated *in vitro*. The results showed that Jas and Cyto D affected the actin distribution in HSC-T6 cells. Treatment with Jas resulted in thick actin bundles and a patchy appearance in the cytoplasm in HSC-T6 cells. In parallel, polymerization of actin microfilaments induced by Jas upregulated the expression of α-SMA and collagen 1, and also enhanced the migration and adhesion properties of HSC-T6 cells. Furthermore, the activation of HSC-T6 cells induced by the reorganization of the actin cytoskeleton was associated with the p38 mitogen-activated protein kinase (p38 MAPK) pathway. In conclusion, the present study suggests that the reorganization of the F-actin cytoskeleton is associated with HSC activation and that the p38 MAPK pathway is involved in this process. The inhibition of F-actin reorganization may thus be a potential key factor or molecular target for the control of liver fibrosis or cirrhosis.

## Introduction

It has been reported that hepatic stellate cells (HSCs) are involved in the development of hepatic fibrosis and in cancer cell invasiveness (1–3). Under physiological conditions, HSCs are quiescent and have important roles in the regulation of retinoid homeostasis and extracellular matrix (ECM) remodeling. However, when the liver is damaged by certain factors, including viral infection, chronic alcohol abuse and inflammation, quiescent HSCs undergo a process of activation that is characterized by trans-differentiation into α-smooth muscle actin (α-SMA)-positive myofibroblast-like cells, and produce a large quantity of ECM components, including collagen types 1 and 3, as well as other matrix proteins. The fibrogenic features of HSCs, together with an induced ability to synthesize and deposit ECM components, represent a key cellular event in the genesis of liver cirrhosis.

The cytoskeleton is accountable for a variety of physiological events in the cell, including the formation of stress fibers, adhesion, migration, apoptosis and receptor clustering in different cell models (4,5). A study by Yee (6) indicated that the activation of HSCs is accompanied by changes in the cellular cytoskeleton. As a highly conserved protein, actin constitutes an essential component of the cytoskeleton in most cells and exists in two principal forms: Globular monomeric (G) and filamentous polymeric (F). G-actin molecules are soluble in diluted salt solution and polymerize into F-actin when their concentration is increased. In culture, HSC activation can be distinguished by the development of prominent cytoplasmic fibers, the loss of perinuclear droplets and cell spreading (i.e., increasing in size). This cytoskeletal reorganization provides the driving force for cell movement and surface remodeling (7). Based on these observations, it was hypothesized in the present study that the actin cytoskeleton is directly involved in the morphological and functional changes in HSCs that are associated with activation. HSC-T6 is a rat hepatic stellate cell line (8) derived from primary HSCs as an *in vitro* assay system. For the establishment of this cell line, the primary HSCs were transformed with the simian virus 40 large T-antigen and a stable phenotype exhibiting an activated phenotype with a fibroblast-like shape and high proliferation activity was established (9). It is considered that the immortalized cells are likely to prove useful in exploring the key process involved in hepatic fibrogenesis. To evaluate this hypothesis, the HSC-T6 cells were treated with either the F-actin stabilizer jasplakinolide (Jas) or the depolymerizer cytochalasin D (Cyto D). The actin cytoskeleton was then evaluated by assessment of stress fiber formation in HSCs. In the present study, the effects of the cytoskeletal reorganization induced by Jas or Cyto D on the activation of HSCs were investigated using a variety of experimental tools.

## Materials and methods

### Cell culture

HSC-T6 cells, a spontaneously immortalized rat HSC line, were purchased from the Cell Bank of Xiangya School of Medicine (Changsha, China), and maintained in high-glucose Dulbecco’s Modified Eagle medium (DMEM; Gibco^®^; Invitrogen Life Technologies, Carlsbad, CA, USA) supplemented with 15% (v/v) fetal bovine serum (FBS; HyClone, Waltham, MA, USA). The study was approved by the Ethics Committee of Weifang Medical University (Weifang, China; permit no. 2013024).

### Cytoskeleton staining

Following being serum-starved for 12 h, HSC-T6 cells were treated with Jas (100 nmol/l) (10) or Cyto D (1 μmol/l) (11) for 30 min. Corresponding control groups received equal volumes of dimethylsulfoxide (DMSO). Following being fixed with 4% paraformaldehyde at 4°C for 30 min, cells were stained with 1.0 μg/ml phalloidin-fluorescein isothiocyanate (FITC) (Enzo Life Sciences, Alexis Biochemicals, San Diego, CA, USA) for 40 min at room temperature. The images were acquired using a fluorescence microscope (Leica, Mannheim, Germany).

### Cell proliferation assay

The 5′-ethynyl-2′-deoxyuridine (EdU) incorporation assay was performed to quantify cell proliferation according to the manufacturer’s instructions (Guangzhou Ribobio Co., Ltd, Guangzhou, China). More than five random fields per well were captured (magnification, ×100) and Image-Pro Plus 6.0 (Media Cybernetics, Inc., Rockville, MD, USA) was used to calculate the percentage of EdU-positive cells identified by Apollo^®^ 567 fluorescence in the total cells identified by Hoechst 33342 nuclear staining.

Cell proliferation was also examined using the cell counting kit-8 (CCK-8, Dojindo Molecular Technologies, Kumamoto, Japan). HSC-T6 cells (1×10^4^ cells/well) were seeded in 96-well plates and incubated overnight in DMEM containing 10% FBS. The cells were then transferred to serum-free conditions for 12 h. Following treatment with Jas or Cyto D, 100 μl medium containing cell counting kit-8 was added to the cells in the 96-well plates, which were subsequently incubated for 2 h at 37°C. The absorbance at 450 nm was determined using a multi-plate reader (Lambda Bio-20; Beckman Coulter, Inc., Brea, CA, USA).

### Cell adhesion assay

Cells were trypsinized and resuspended in serum-free media containing 0.25% bovine serum albumin. Equal numbers of cells were seeded onto the plates and incubated for 1 h at 37°C. Following the removal of non-adherent cells by washing, adherent cells were counted independently in six random, high-power microscope fields (HPFs) (magnification, ×100)/well by three observers blinded to the treatments.

### Cell migration assay

A modified Boyden chamber (Costar, Cambridge, MA, USA) assay was used to evaluate the migratory function of cells. Briefly, a total of 1×10^5^ HSC-T6 cells were placed in the upper chamber, while the medium was placed in the lower chamber. The assays were conducted over a 16-h incubation period at 37°C in an incubator equilibrated with 5% CO_2_. The membrane was then gently washed with PBS and fixed with 4% paraformaldehyde. Non-migrating cells were gently removed with cotton balls from the upper side of the membrane, and the membrane was then stained with DAPI. The migration of late HSCs was evaluated by counting the migrated cells in six random HPFs (magnification, ×100)/well.

### Cell apoptosis assay

HSC-T6 cells (1×10^6^) were stained with annexin V-FITC and propidium iodide (PI) (BD Biosciences, Franklin Lakes, NJ, USA). Following staining, the cells were washed twice with binding buffer. Apoptotic cells were detected by fluorescence-activated cell sorting (FACS). Fluorescence parameters were gated using unstained and single-stained cells, and 20,000 cells were collected for each sample. Apoptotic percentage analysis was performed using CellQuest™ software (BD Biosciences).

### RNA isolation and quantitative polymerase chain reaction (qPCR)

Total cellular RNA was isolated using TRIzol^®^ reagent (Invitrogen Life Technologies) and reverse-transcribed into cDNA using the SYBR^®^ PrimeScript^®^ RT-PCR kit (Takara Bio, Inc., Shiga, Japan) at 37°C for 15 min. Gene expression was evaluated using SYBR^®^ Premix Ex Taq™ (Takara). The rat α-SMA sequences were: Forward, AGCCAGTCGCCATCAGGAAC, and reverse, CCGGAGCCATTGTCACACAC. The collagen type 1 sequences were: Forward, GACATGTTCAGCTTTGTGGACCTC, and reverse, AGGGACCCTTAGGCCATTGTG. GAPDH was used as an internal control, and the sequences were: Forward, GGCACAGTCAAGGCTGAGAATG, and reverse, ATGGTGGTGAAGACGCCAGTA. The thermal cycling conditions were as follows: 30 sec at 95°C for pre-denaturation, followed by 42 cycles of 15 sec at 95°C for denaturation, 1 min at 59°C for annealing and 10 sec at 72°C for elongation. At the end of each cycle, the fluorescence emitted by SYBR^®^ Green I was measured. Following completion of the cycling process, samples were immediately subjected to a temperature ramp for melting curve analysis. Relative gene expression was analyzed with the comparative Ct method (2^−ΔΔCt^).

### Western blot analysis

Proteins were subjected to 12% SDS-PAGE and then transferred onto a polyvinylidene fluoride membrane. Following blocking in 5% milk in Tris-buffered saline with Tween 20 (TBST), the membranes were treated with primary antibodies against α-SMA (Sigma, St. Louis, MO, USA), collagen type 1 (Santa Cruz Biotechnology, Inc., Santa Cruz, CA, USA) and phosphorylated-p38 mitogen-activated protein kinase (phospho-p38 MAPK; Cell Signaling Technology, Inc., Danvers, MA, USA; 1:100 dilution). GAPDH (Santa Cruz, USA) or p-38 MAPK (Cell Signaling Technology, Inc., Danvers, MA, USA, 1:100 dilution) were used as control measures. Membranes were then washed with TBST and incubated with secondary antibody conjugated to horseradish peroxidase (Santa Cruz Biotechnology, Inc., 1:2,000 dilution). Immunoreactive bands were visualized by enhanced chemiluminenscence (Amersham Pharmacia Biotech, Amersham, UK), and the resulting autoradiograms were analyzed by densitometry.

### Statistical analysis

Unless otherwise indicated, results are expressed as the mean ± standard error from 3–5 independent experiments. Statistical analyses were performed using one-way analysis of variance, followed by Tukey’s test for inter-group comparisons. P<0.05 was considered to indicate a statistically significant difference. All data were analyzed using SPSS 15.0 software (SPSS, Inc., Chicago, IL, USA).

## Results

### Effects of Jas or Cyto D on the actin cytoskeleton reorganization in HSC-T6 cells

**Figure 1:**
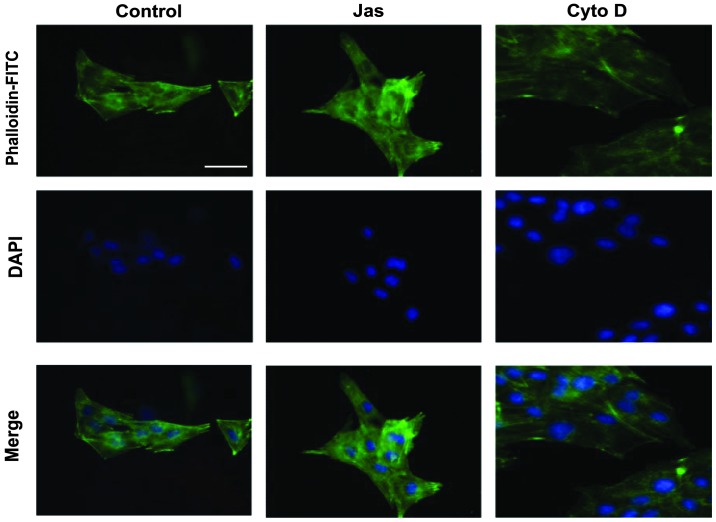
Effects of Jas or Cyto D on the actin cytoskeleton reorganization in HSC-T6 cells. HSC-T6 cells were incubated with dimethylsulfoxide (vehicle control), Jas (100 nmol/l) or Cyto D (1 μmol/l), respectively, for 1 h prior to being fixed in 4% (v/v) paraformaldehyde for 15 min. The actin filaments were then stained with phalloidin-FITC and the nuclei with DAPI. Images were captured using a fluorescence microscope. Scale bar, 50 μm; magnification, ×400. FITC, fluorescein isothiocyanate; Cyto D, cytochalasin D; Ja, jasplakinolide.

To evaluate the effects of Jas or Cyto D on the actin reorganization in HSC-T6 cells, the distribution of stress fibers, which can be easily detected by phalloidin, was observed under the fluorescence microscope. The distribution of F-actin in DMSO (vehicle control)-treated HSC-T6 cells exhibited a small network of parallel stress fibers. Treatment with 100 nmol/l Jas for 1 h resulted in thick actin bundles and a patchy appearance in the cytoplasm. By contrast, Cyto D (1 μmol/l)-treated cells typically exhibited dissolution of actin stress fibers and decreased fluorescent staining (Fig. 1).

### Comparative effect of the reorganization of the actin cytoskeleton induced by Jas or Cyto D on HSC-T6 cell functions

**Figure 2:**
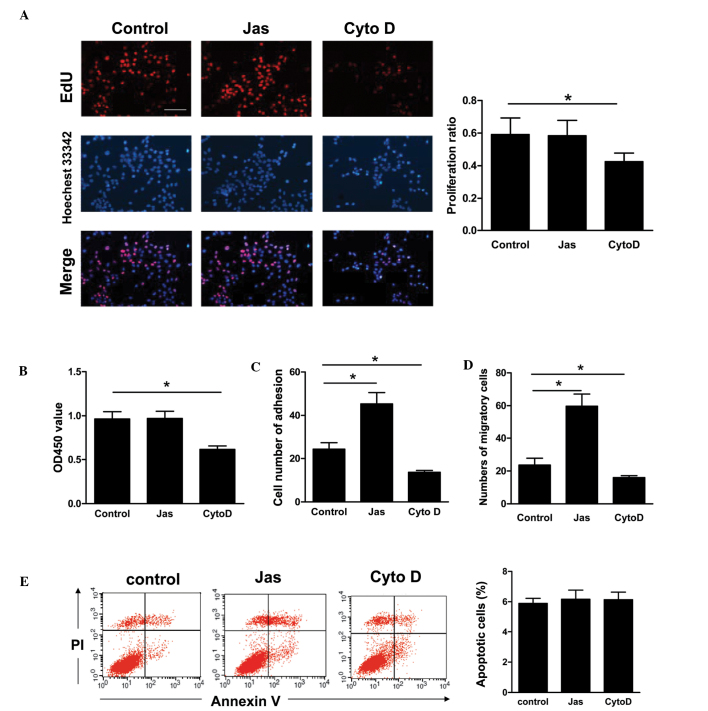
Comparative effect of actin cytoskeleton reorganization induced by Jas or Cyto D on HSC-T6 cell functions. (A) HSC-T6 cells were incubated with DMSO, Jas (100 nmol/l) or Cyto D (1 μmol/l), respectively, for 1 h. Cells were then washed and cultured in fresh medium for 12 h. Cell proliferation was detected by the EdU incorporation assay. At least seven random fields from each well were captured (scale bar, 100 μm, magnification, ×100), and Image-Pro Plus 6.0 was then used to calculate the percentage of EdU-positive cells in the sample. (B) Cell proliferation was assessed using the cell counting kit-8 assay. (C) Following pretreatment with DMSO, Jas or Cyto D, HSC-T6 cells were re-seeded into plastic wells for 1 h at 37°C. Following removal of nonadherent cells by washing with phosphate-buffered saline, adherent cells were counted and analyzed. (D) Cell migration was tested in a modified Boyden chamber assay. Following treatment, HSC-T6 cells (1×10^5^) were placed in the upper layer. The lower chamber was filled with medium and incubated for 16 h. The migrated cells were stained with DAPI and analyzed. (E) The apoptotic cells were quantified by fluorescence-activated cell sorting following annexin V-FITC and PI staining. Annexin V-positive and PI-negative cells were defined as apoptotic cells. Data are expressed as the mean ± standard error of three different experiments. ^*^P<0.05. FITC, fluorescein isothiocyanate; Cyto D, cytochalasin D; Jas, jasplakinolide; PI, propidium iodide; EdU, 5′-ethynyl-2′-deoxyuridine; DMSO, dimethylsulfoxide; OD, optical density.

The effects of the actin cytoskeleton reorganization induced by Jas or Cyto D on the HSC-T6 cell functions were investigated in a series of studies (Fig. 2). Initially, cell proliferation was evaluated using the CCK-8 and EdU incorporation assays. Compared with the DMSO-treated HSC-T6 cells, cell proliferation was decreased in the Cyto D-treated group. However, exposure to Jas did not significantly affect the proliferative activity of HSC-T6 cells (Fig. 2A and B). Furthermore, the adhesion and migration of HSC-T6 cells were determined using the adhesion assay and a modified Boyden chamber, respectively. The results showed that Jas increases, but Cyto D impairs, the adhesion and migration of HSC-T6 cells (Fig. 2C and D). Additionally, apoptotic cells (annexin V^+^/PI^−^) were detected by FACS. The percentages of apoptotic HSC-T6 cells in the Jas- or Cyto D-treated groups were observed to be similar to those in the DMSO-treated group (Fig. 2E).

### Effect of the reorganization of the actin cytoskeleton induced by Jas or Cyto D on the activation of HSC-T6 cells

**Figure 3:**
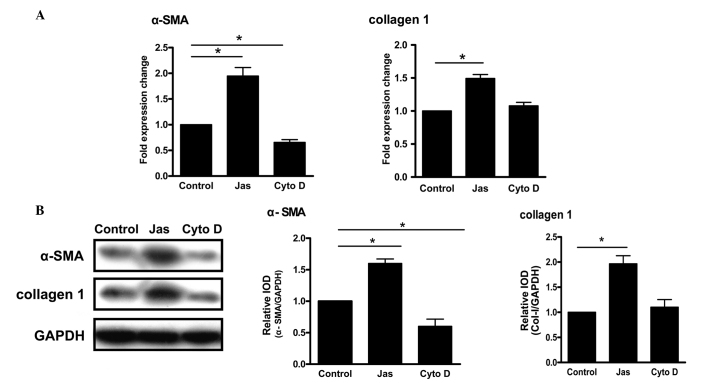
Effect of the actin cytoskeleton reorganization induced by Jas or Cyto D on the activation of HSC-T6 cells. (A) HSC-T6 cells were treated with dimethylsulfoxide, Jas or Cyto D, respectively. The gene expression of α-SMA and collagen type 1 was assessed by the quantitative polymerase chain reaction. Data were analyzed using the 2^−ΔΔCt^ method. (B) The protein expression of α-SMA and collagen type 1 was assessed by western blot analysis. Following treatment, cell lysates were resolved using 12% SDS-PAGE, followed by transfer to a polyvinylidene fluoride membrane. Western blot analysis was performed with specific antibodies and each band was detected using enhanced chemoluminescence reagent. Data are expressed as the mean ± standard error of five experiments. ^*^P<0.05. α-SMA, α-smooth muscle actin; IOD, integrated optical density; Cyto D, cytochalasin D; Jas, jasplakinolide.

Increased expression levels of α-SMA and collagen type 1 are considered to be the major markers of HSC activation (12,13). Compared with the control group, treatment with Jas increased the mRNA levels of α-SMA and collagen type 1. By contrast, the gene expression of α-SMA in the Cyto D-treated group was lower than that in the control group (Fig. 3A). Similar results were obtained for the protein levels (Fig. 3B).

### Actin cytoskeleton reorganization-induced HSC-T6 cell activation is associated with the p38 MAPK pathway

**Figure 4:**
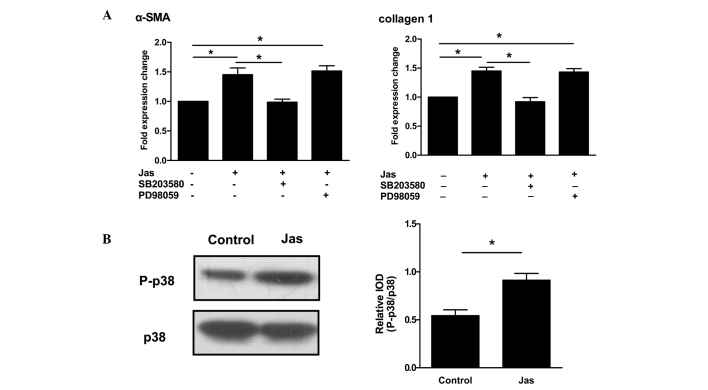
Actin cytoskeleton reorganization-induced HSC-T6 cell activation correlates with the p38 MAPK pathway. (A) HSC-T6 cells were pretreated with PD98059 (10 μmol/l) or SB203580 (100 nmol/l), respectively, for 30 min. The cells were then exposed to DMSO or Jas. Gene expression of α-SMA and collagen type 1 was then determined using the quantitative polymerase chain reaction. (B) The phosphorylation status of p-38 MAPK was assessed by western blot analysis. HSC-T6 cells were incubated with DMSO or Jas for 1 h. Cell lysates were resolved using 12% SDS-PAGE, followed by transfer to a polyvinylidene fluoride membrane. Western blot analysis was performed with specific antibodies to distinguish between different phosphorylation statuses of p38 MAPK. In addition, total p38 MAPK was analyzed as a loading control. P-p38 MAPK was densitometrically analyzed and normalized to total p38 MAPK. The results are expressed as the mean ± standard error of five experiments. ^*^P<0.05. α-SMA, α-smooth muscle actin; IOD, integrated optical density; Jas, jasplakinolide; DMSO, dimethylsulfoxide; p38 MAPK, p38 mitogen-activated protein kinase; P-p38, phosphorylated p38 MAPK.

Both extracellular signal-regulated kinase (ERK) and p38 MAPK have been shown to regulate HSC activation (14,15). To explore whether those signaling molecules were involved in the actin cytoskeleton reorganization-induced HSC activation, HSC-T6 cells were pre-incubated for 30 min with inhibitors of ERK (PD98059) or p38 MAPK (SB203580) prior to treatment with Jas. qPCR revealed that PD98059 did not affect the expression of α-SMA or collagen type 1 induced by Jas. By contrast, inhibition of p38 MAPK by SB203580 significantly reduced the Jas-induced expression of α-SMA and collagen type 1 (Fig. 4A).

Since p38 MAPK is critical for the actin cytoskeleton reorganization-induced HSC activation, its activation in HSC-T6 cells treated with Jas was further evaluated. Western blot analysis showed that Jas significantly increased the protein levels of phospho-p38 in HSC-T6 cells (Fig. 4B).

## Discussion

As described previously in this study, during the development of liver fibrogenesis, HSCs undergo a response known as activation, which is the transition of quiescent cells into proliferative, fibrogenic and contractile myofibroblasts (16). However, the mechanism by which HSCs are activated is unclear. Recently, a study by Rombouts *et al* (5) indicated that the acquisition of certain properties by activated HSCs is highly dependent on the reorganization of the actin cytoskeleton (4). The present study provided direct evidence showing that changes in the actin cytoskeleton, including the assembly of stress fibers, affects the activation of HSCs. As shown in Fig. 1, the F-actin staining of HSCs showed a variation in the intracellular distribution of F-actin between cells incubated with Jas and those incubated with Cyto D. Jas treatment resulted in thick actin bundles and a patchy appearance in the cytoplasm of HSCs. By contrast, Cyto D-treated cells typically exhibited dissolution of actin stress fibers and decreased fluorescent staining. In parallel, the polymerization of actin microfilaments by Jas led to HSC activation: i) Jas upregulated the expression of α-SMA and collagen type 1; ii) stabilization of F-actin by Jas improved the migration and adhesion properties of HSCs. Furthermore, the HSC-T6 cell activation induced by the actin cytoskeleton reorganization was associated with the p38 MAPK pathway.

Ikeda *et al* (17) demonstrated that an aberrant actin cytoskeleton in mice deficient for destrin (an actin depolymerizing factor) was able to cause cell hyperproliferation. Furthermore, the ability of the ECM to modulate cell growth may be mediated partly by the assembly and disassembly of F-actin filaments (18). Although exposure to Jas did not significantly affect the proliferative activity of HSCs in the present study, the cell proliferation was decreased in the Cyto D-treated group. The results also indicate that F-actin has an important role in the regulation of HSC proliferation.

It is well accepted that F-actin is crucial in determining cell shape and migration, as well as in controlling apoptosis (19). Consistent with this, the stabilization of actin by Jas in the present study was observed to significantly augment the migration and adhesion of HSCs, which, by contrast, were impaired by Cyto D. However, F-actin rearrangement appeared to have no effect on the apoptosis of HSCs.

Since the increased expression of α-SMA is considered to be a major marker of the activation of HSCs, the association between F-actin and the expression of α-SMA was examined. The stabilization of actin by Jas significantly increased the expression of α-SMA in HSCs, indicating that F-actin is involved in the activation process of HSCs. Activated HSCs are known to increase the synthesis/secretion of fibrogenic ECM components, including collagen types 1, 3, 5 and 6 (20–22). A study has reported that the expression of collagen type 3 is more significant at the early stages of fibrosis (mild activated HSCs), while collagen type 1 is more significant at the advanced stages (intensive activated HSCs) (23). In the present study, it was found that the activation of HSCs promoted via the stabilization of actin by Jas produced increased levels of collagen type 1. It was thus confirmed that the polymerization of F-actin led to the activation of HSCs. Moreover, the activation of HSCs induced by cytoskeletal reorganization was found to be dependent on p38 MAPK, since SB203580, a p38 MAPK inhibitor, inhibited the gene and protein expression of α-SMA and collagen type 1 induced by treatment with Jas.

The findings of the present study may be of importance since they not only indicate that actin reorganization has a pivotal role in regulating the biological functions of HSCs, including cell adhesion and migration, but also provide further insights into the possible molecular mechanisms behind the activation of HSCs. The inhibition of F-actin reorganization may thus be a key factor or molecular target for the control of liver fibrosis or cirrhosis.
